# Advances in Wound Healing Strategies for Diabetic Foot Ulcers: From Conventional to Regenerative Therapies

**DOI:** 10.2174/0115733998407601251010114114

**Published:** 2025-11-25

**Authors:** Abdullah Alwahbi

**Affiliations:** 1 Vascular Surgery, King Saud bin Abdulaziz University for Health Sciences, Riyadh, Saudi Arabia

**Keywords:** Diabetic foot ulcer, pathophysiology, wound healing, conventional therapies, novel strategies, risk of amputation and mortality

## Abstract

Diabetic Foot Ulcer (DFU) is a serious and chronic complication of uncontrolled diabetes, significantly increasing the risk of amputation and mortality in diabetic patients. Conventional wound healing techniques often fail to manage chronic wounds in DFU due to several limitations, which eventually raises the need for advanced, innovative, and efficient treatment strategies for the better management of DFU. Many novel interventions, such as growth factor therapy, stem cell therapy, gene therapy, nanotechnology, bioengineered skin substitutes, 3D-bioprinting, and regenerative medicine, have shown promising roles in wound healing and tissue regeneration, suggesting their potential implications in the treatment and control of DFU. An integrated and multidisciplinary approach was proven to be a promising option for rapid and effective DFU management, eventually reducing the financial burden on the patients and healthcare system. Besides, AI-driven medical technologies were found to support the healthcare system for the prevention, prediction, diagnosis, and personalized treatment of DFU. The present review article provides a brief overview of DFU and its pathophysiology. It highlights traditional DFU treatment strategies, their limitations, and focuses on emerging innovative strategies, emphasizing their potential for addressing DFU. Moreover, it sheds light on challenges and future research areas of these emerging interventions.

## 1 INTRODUCTION

Diabetic foot ulcer (DFU) is one of the most severe complications of uncontrolled and persistent diabetes, which increases the risks of morbidity and mortality [1]. According to the International Working Group on the Diabetic Foot (IWGDF), DFU is characterized by infection, ulceration, or tissue damage in the foot, commonly associated with neuropathy and/or peripheral artery disease in individuals with diabetes mellitus [2]. Recent studies highlight the role of a triad of pathological mechanisms comprising peripheral neuropathy, vascular disease/insufficiency, and secondary bacterial infection in DFU development [1, 3]. Besides, prolonged diabetes, gender, aging, impaired healing, alteration of the extracellular matrix (ECM), comorbidities, and family history of DFU are other important risk factors of DFU [4]. Diabetes affects more than 550 million people globally, and every year, around 18.6 million diabetic people with diabetes develop DFU, leading to severe complications, eventually increasing the risk of death [1, 3, 5]. Around 19-34% of diabetic patients have a lifetime risk of developing DFU. Among those affected, 5% of DFU patients undergo amputations, while an alarming 50-70% succumb to the condition within 5 years [6]. DFU is associated with life-threatening complications, including diabetic ketoacidosis, hyponatremia, septic shock, foot deformity, callus deformity, plantar shear stress, Charcot neuroarthropathy, osteomyelitis, and risk of amputation [7, 8].

The extremely high cost of DFU treatment imposes a significant financial burden on patients and healthcare systems [9, 10]. Conventional wound healing techniques often struggle to manage chronic wounds in DFU patients effectively [11]. Therefore, there is a growing global need for innovative and advanced wound healing strategies that promote tissue regeneration, accelerate wound healing, and reduce the risk of infection in DFU. This review article provides a comprehensive overview of DFU with the pathophysiology of wound healing. Besides, we have discussed conventional DFU treatments and their limitations, and we have critically reviewed several emerging innovative wound healing strategies for DFU management. Further, we explored the role of regenerative medications in DFU treatment and concluded with the challenges and prospects of these emerging innovative wound healing strategies.

## 2 METHODOLOGY

For this review, an online extensive literature search was conducted using key terms such as “diabetic foot ulcers,” “pathophysiology of wound,” “wound healing,” “conventional wound healing of DFU,” “debridement,” “wound dressing,” “offloading,” “antibiotic therapy,” “advanced therapies for DFU,” and “regenerative therapy for DFU” on PubMed and Google Scholar to find pertinent academic articles published in English language between the time period 2003 and 2025. Reference lists of selected articles were also analysed for additional and important studies.

## 3 PATHOPHYSIOLOGY OF WOUND HEALING IN DFU

Normal wound healing is a complex, coordinated process involving immune cells, cellular factors, extracellular components, growth factors, and chemotactic agents, and categorized into four stages: hemostasis, inflammatory response, tissue regeneration and proliferation, and tissue remodelling [12, 13]. Impaired, and delayed wound healing is a common complication in diabetic patients due to disruption of the stages of wound healing triggered by hyperglycemia, hypoxia, persistent inflammation, neuropathy, elevated levels of reactive oxygen species (ROS), and matrix metalloproteinases (MMPs), recurrent infections, and damaged neuropeptide signalling, often leading to DFU [12, 14-18]. DFU wounds have a longer inflammatory phase than non-diabetic wounds, which delays healing and leads to chronic wounds, highlighting inflammation's significant role [19, 20]. Although a low level of ROS is essential for cell signalling and wound healing, the overproduction of ROS in DFU leads to increased oxidative stress, causing damage to cells and tissue, leading to chronic wounds [13, 21-24]. In addition, vascular dysfunction, characterized by impaired blood vessel function— including abnormalities in relaxation, contraction, microvascular regulation, endothelial function, arterial stiffness, and neural activities —plays a critical role in the development of DFU [25-27].

One of the key complications in DFU is the presence of diverse microbiota and the formation of biofilms at the wound site, which delay healing, contribute to chronic infections, and complicate treatment [28, 29]. DFU is generally polymicrobial, comprising a wide range of microbes that can coexist, either in a planktonic or sessile state, ranging from aerobic to anaerobic bacteria as well as fungi [29, 30]. Microbes in biofilms shield themselves from the host's immune response and antibiotic treatments, leading to the emergence of multi-drug resistant (MDR) strains. This diminishes the effectiveness and even the rejection of therapeutics, consequently leading to chronic infection in DFU [31, 32].

## 4 CONVENTIONAL WOUND HEALING APPROACHES

The conventional DFU managements involve surgical methods like debridement, skin grafts, and flaps, while non-surgical approaches include wound dressing, skin substitutes, and topical formulations.

## 5 DEBRIDEMENT TECHNIQUES

Debridement is the principal step in DFU treatment which involves removal of necrotic, hyperkeratotic dermal tissue, foreign and infected debris from the wound bed to accelerate the wound healing by stimulating local growth factors, promoting granulation, regeneration, re-epithelialization, wound drainage and reducing bacterial count, blood pressure, and amputation risk [4, 33-35]. Debridement techniques vary, including surgical (sharp), mechanical, enzymatic, autolytic, and biological, each with distinct advantages and limitations. Surgical debridement is recognized as the cost-effective and standard treatment strategy for wound healing in DFU. It involves the excision of necrotic and infected tissue, followed by the application of saline-moistened cotton gauze, and requires a skilled healthcare professional [33, 34, 36]. Mechanical debridement technique involves the removal of hardened necrotic tissue using wet-to-dry dressings, high-pressure irrigation, pulsatile lavage, and hydrotherapy. While effective, it non-specifically removes both necrotic and regenerating tissue, causing patient pain. Enzymatic debridement is a systemic but expensive technique that uses enzyme formulations for rapid digestion of proteins, fibrins, elastin, and collagen in necrotic wound exudate. Autolytic debridement is a minimally invasive method that promotes a moist environment in wounds to accelerate the function of enzymes and the shedding of necrotic tissue naturally. It is a time-consuming, cost-effective technique, beneficial for painful wounds [33, 37]. The biological debridement includes larval therapy, where green bottle fly (*Lucilia sericata*) larvae are applied to wounds, which debride, disinfect, and digest necrotic tissue by secreting enzymes and promote healing. This method selectively targets damaged tissue but is expensive [33, 38].

## 6 WOUND DRESSINGS

Wound dressings are vital for wound healing and play a key role in DFU management [39, 40]. Biocompatible and biodegradable modern dressings such as hydrogels, foams, alginates, and antimicrobial agents effectively promote healing and manage DFU by retaining moisture, enhancing hypoxia, and reducing pain [33, 40]. Hydrogels are 3D-networks of hydrophilic substances formed as sheets, amorphous structures, or beads that rehydrate wounds by absorbing exudate [33]. These are biocompatible, less toxic, and have excellent fluid handling potential. They promote autolysis of necrotic tissue, cell proliferation, angiogenesis, and inhibit bacterial growth in DFU [39-43]. Foam dressings made of polyaniline, polyurethane, or silicone, either hydrophilic or hydrophobic, act as bacterial barriers and provide protection and thermal insulation to wounds, retain moisture, and inhibit biofilm formation in DFU [33, 39, 40]. Foam dressings are preferred for their low cost, absorbency, pliability, ease of use, and antimicrobial delivery for managing DFU [44-46]. Alginate-based dressings are thin fibrous sheets made up of sodium alginate, calcium alginate, or a combination of both. They are highly absorbent, non-adherent, bacteriostatic, and form a gel upon contact with the wound surface, retaining moisture and reducing bacterial infections [39, 40, 47]. Different types of antimicrobial dressing impregnated with various microbiocidal compounds, including antibiotics, honey, essential oils, iodine, chitosan, chlorhexidine or polyhexamethlene biguanide, silver, and nanoparticles, are used due to their antimicrobial, anti-inflammatory, antiseptic properties to treat wounds for the accelerated wound healing [39, 48-51].

## 7 OFFLOADING STRATEGIES

Offloading is crucial for treating neuropathic plantar foot ulcers in DFU patients as per the IWGDF guidelines, reporting non-removable off-loading as more effective than removable off-loading for rapid and better wound healing [6, 34, 52, 53]. The removal or redistribution of plantar pressure and the protection of pressure points on the foot can be achieved through offloading with various tools, including total contact casts, therapeutic footwear, orthoses, foams, and felt padding. [4, 6, 33, 35]. Total contact casting is a non-removable, rigid, knee-high offloading device consisting of padding and casting material. It immobilizes joints and soft tissue while protecting wounds from friction, pressure, and trauma by redistributing plantar pressure, thus reducing pain in DFU patients [51, 54, 55]. Therapeutic footwear is a removable off-loading device recommended by the IWGDF for high-risk patients (IWGDF risk 2-3) to reduce DFU by preventing friction, lowering plantar pressure, and shear forces [53, 56, 57].

## 8 ANTIBIOTIC THERAPY FOR INFECTION MANAGEMENT

Antibiotic therapy is essential for controlling biofilm formation and infections in DFU wounds, but it depends on microbiological findings and antibiotic resistance [6]. Various broad-spectrum antibiotic combinations, such as ampicillin and sulbactam, ticarcillin and clavulanic acid, piperacillin and tazobactam, clindamycin with cefoperazone-sulbactam or piperacillin-tazobactam, and cefepime with gentamicin, imipenem, amikacin, and tazobactam, are used for the management of moderate to severe infections [58]. Topical antibiotic treatment involves application of antibiotics directly to the wound surface or through dressings to prevent and treat mild infections of DFU [59]. A variety of antibiotics, including gentamicin, mupirocin, bacitracin, vancomycin, neomycin, chloramphenicol, and polymyxin B, have been used for topical therapy to treat wounds in DFU and eradicate polymicrobial biofilms [60, 61]. Systemic antibiotic treatment involves administering antibiotics either orally (for superficial ulcers with mild infection) or intravenously (for deep, chronic infections) to treat wounds. Regular use of antibiotics helps control infections in DFU and reduces the risk or extent of amputations [6, 59].

## 9 ADVANCED THERAPIES IN DFU MANAGEMENT

Conventional treatments for DFU are inadequate, increasing the need for cost-effective advanced therapies globally to lower morbidity, mortality, and financial strain [36]. New technology has led to innovative wound treatment methods, including moist dressings, responsive dressings, growth factor therapies, stem cell therapy, bioengineered skin substitutes, gene therapy, nanotherapeutics, and 3D bioprinting (Table **1**) [33, 36, 62, 63].

## 10 GROWTH FACTOR-BASED THERAPIES

Growth factor-based therapies utilize multifunctional bioactive polypeptides at the wound site to accelerate repair and enhance the healing process by promoting cell migration, epithelialisation, angiogenesis, cell proliferation, differentiation, matrix synthesis, and remodelling. They are also involved in modulating inflammation, reducing scar formation, lowering the risk of wound infections, and enhancing blood, oxygen, and nutrient supply to the healing tissue [62, 63]. Commonly recognized growth factors comprise platelet-derived growth factor (PDGF), vascular endothelial growth factor (VEGF), epidermal growth factor (EGF), fibroblast growth factor (FGF), transforming growth factor beta (TGFβ), hepatocyte growth factor (HGF), insulin-like growth factor (IGF), and stromal cell-derived factor 1 (SDF-1), all of which are found to accelerate wound healing and tissue repair in DFU by enhancing chemotaxis and mitogenesis of different cell types (neutrophils, fibroblasts, monocytes) [62, 64].

## 11 STEM CELL THERAPY

Stem cell therapy is one of the novel advanced therapeutic strategies that utilize stem cells to enhance the chronic wound healing process and restore normal skin architecture. This is due to their long-lasting self-renewal properties and potential to differentiate into multiple cell types [36, 65]. Stem cell-based therapy employs various processes, including angiogenesis, the release and interaction of growth factors, immunomodulation, stimulation of vascular differentiation, re-epithelialization, inflammation regulation, and collagen deposition [36, 66, 67]. Several types of stem cells, including mesenchymal stem cells (MSCs), induced pluripotent stem cells (iPSCs), embryonic stem cells (ESCs), and adipose-derived stem cells (ASCs), serve as the primary resources for skin regeneration and wound healing [65]. Among them, MSCs are involved in all stages of wound healing, promoting cell migration, angiogenesis, re-epithelialisation, and the release of growth factors and cytokines, which leads to accelerated wound closure. Similarly, ASCs are highly abundant and easily obtainable for wound healing [36, 65, 68, 69]. ESCs could be the most effective stem cells for tissue regeneration and wound healing due to their potential for self-renewal and differentiation. Still, ethical and legal aspects have limited their use in clinical investigations. iPSCs represent a modern class of pluripotent stem cells that play a promising role in tissue regeneration, wound healing, and blood vessel enhancement [65, 67]. Several clinical and preclinical investigations have reported the beneficial impact of stem cells in the wound healing of DFU by the release of cytokines, growth factors, and promoting skin regeneration [69-72].

## 12 TISSUE ENGINEERING AND BIOENGINEERED SKIN SUBSTITUTES

The bioengineered skin substitutes (BSS) are tissue-engineered skin grafts or skin analogs displaying human skin-like biological and pharmacological properties and emerged as a novel alternative wound care strategy [73]. BSS protects wounds by promoting the inflow of fibroblasts, neutrophils, macrophages, keratinocytes, and endothelial cells into the wound bed. It also secretes cytokines and growth factors that ultimately stimulate angiogenesis, re-epithelialization, the deposition of ECM, tissue regeneration, reducing bacteria, and preventing fluid loss [74]. BSS provides a scaffold for growth, angiogenesis, tissue regeneration, proliferation, and immunomodulation, and plays a crucial role in the wound healing of DFU. Based on their content and number of layers, BSS are categorized into four major types: Acellular single layer, Cellular single layer, Acellular Bilayer, and Cellular Bilayer [73]. Several investigations have reported different types of BSS and their promising potential in the DFU management [73, 75, 76].

## 13 GENE THERAPY APPROACHES

Gene therapy represents a novel and promising approach for managing chronic wounds, which fall into three categories: gene augmentation, gene silencing, and gene editing [77]. This approach involves the efficient incorporation of genetically modified cells or the delivery of vectors with properly functioning genes to the wound site. This method addresses impaired functions of damaged cells or defective/mutated genes and promotes rapid wound healing [78]. The *ex vivo* gene therapy involves the direct incorporation of desired functional genes into specific cell types obtained from patients, followed by the reintroduction of modified cells into the patient. In contrast, *in vivo* gene therapy involves directly introducing genes into the target tissue of the patient using vectors that contain the desired gene. Several studies have reported effective applications of angiogenic growth factor-based gene therapies for the treatment of diabetic foot ulcers *via* promotion of chemotaxis, angiogenesis, epithelialization, and collagen production [79-82].

## 14 NANOTECHNOLOGY-BASED STRATEGIES

The technological advancement in the field of biomedicine and nanotechnology accelerated the effective use of nanomaterials in the form of nanoparticles, nanoemulsions, nanoliposomes, nanofibers, and hydrogels for wound healing by their involvement in the different wound healing processes and antibacterial activity [36, 64, 83, 84]. Moreover, these methods provide other advantages, such as cell specificity, topical drug delivery, increased penetration, controlled release of drugs, and better interactions [36, 64, 83]. Metallic nanoparticles (silver, gold, zinc, gallium and oxides of zinc, copper, aluminium, titanium, and iron), polymeric nanomaterials and nitric oxide, cerium, bioactive glass, and carbon-based nanoparticles have been extensively used in the treatment of chronic wounds due to their inherent bioactivities, antibacterial and wound healing properties [36, 85]. In addition, nanoparticle-based products such as nanoemulsions, nanoliposomes, nanofibers, and smart hydrogels, whether used alone or encapsulating growth factors, peptides, antibiotics, and extracellular substrates, deliver enhanced wound healing and protection [64, 83]. Besides, nanofiber scaffolds have been used for enhancing cell adhesion and proliferation by mimicking the ECM of the skin [63].

## 15 3D BIOPRINTING FOR WOUND REGENERATION

3D-bioprinting is an emerging high-throughput automation technology that involves the design and synthesis of biocompatible tissues or organs or skin using bio-ink comprising cells, biomolecules, growth factors, and biomaterials *via* layer-wise deposition, which plays a significant role in wound healing through the regeneration approach [36, 56, 86-88]. Bioinks are made of either natural material (alginate, collagen, chitin, chitosan, cellulose, hyaluronic acid, and thrombin) or synthetic materials (polylactic acid, polyglycolic acid, trimethylene carbonate, polycarbonates, polyurethans, ε-caprolactone, polypropylene fumarate, and polyethyleneglycol methacrylate) [89]. Specifically, the extrusion-based bioprinting (EBB) technique is primarily utilized for skin bioprinting due to its various advantages over the other types [36]. Numerous studies have validated the potential applications of 3D-bioprinted skin grafts in wound healing for the management of DFU [56, 86, 88, 90, 91].

## 16 ROLE OF REGENERATIVE MEDICINE IN DFU TREATMENT

Regenerative wound healing encompasses emerging and advanced biomedical technologies, including cutting-edge dressings, bioactive biomaterials, stem cell therapies, growth factors, bioengineered skin grafts, and gene therapies [63]. Regenerative medicine is an advanced treatment strategy that specifically addresses the restoration of impaired functions through the repair and replacement of cells, tissues, or organs, targeting different stages of wound healing [62, 92]. Regenerative medicines comprise customizable matrices, tissue-engineered biomaterials, growth factors, peptides, proteins, small molecules, RNA interferences, autologous and allogeneic cells, and co-delivery of bioactive molecules [78, 93, 94].

Regenerative therapies with biomaterials involve wound healing using natural or synthetic biomaterials by immune modulation, vascularization, synthesis of ECM, and cell infiltration along with providing a moist environment and protection to the wound [62, 78]. * Ex vivo and *in vivo** gene therapy for tissue repair involves efficiently delivering modified cells or vectors with properly functioning desired genes to the wound site of patients to address defective or mutated genes and enhance function [78]. Regenerative medicines based on growth factors or non-coding RNAs (especially, miRNA, lncRNA and siRNA) or peptides or proteins have been reported to have prominent role in inflammation, cell migration, proliferation, differentiation, epithelialization, angiogenesis, matrix synthesis, cell development, metabolism, and remodelling and thereby accelerate the wound healing [63, 95-97]. However, the efficiency, safety issues, lack of understanding of the mechanism, treatment cost, and selection of the best cell type, material, and gene delivery systems are the major obstacles and challenges that limit the application of regenerative medicines [78, 83, 98, 99].

## 17 CHALLENGES AND FUTURE PERSPECTIVES

The advanced and innovative therapies developed for DFU management encounter numerous obstacles to their adoption and implementation in clinical practice due to several reasons. Most of these advanced strategies and tools are very expensive and require extensive training, infrastructure, and maintenance costs, which ultimately limit their applications. Moreover, getting regulatory clinical approvals for these advanced therapies is extremely challenging due to strict government legislation and regulations [36, 100]. However, through artificial intelligence (AI)- driven medical technologies supporting the 4P-model strategy, improved diagnostic accuracy, constant monitoring, effective treatments, and personalized patient care can be achieved [101-103]. Furthermore, new multidisciplinary approaches *via* integration of AI, machine learning (ML), and wearable technologies enable personalized medications and treatment plans for the prevention and therapeutic management of DFU by analysing and processing extensive patient data, including genetic information, lifestyle choices, and medical histories and provide suitable intervention targets and strategies [102-106]. The future of regenerative medicine relies on advancements in medical science, genetic engineering, disease modelling, and AI [92, 99]. By addressing current gaps in research and regulations, future studies must be undertaken with emphasis on cost-effectiveness, integrated wound care, long-term treatment outcomes, and validation of AI’s clinical impact.

## CONCLUSION

DFU poses a serious complication of uncontrolled diabetes, significantly elevating morbidity and mortality risks. Conventional therapies often fail to effectively manage DFU, highlighting the need for novel interventions. These include growth factor therapy, stem cell therapy, bioengineered skin substitutes, gene therapy, nanotechnology, 3D-bioprinting, and regenerative medicine, which show promise in chronic wound healing and tissue regeneration. Multidisciplinary approaches are essential for effective DFU management and reducing the economic burden on patients. Future research should focus on developing cost-effective strategies, integrating interdisciplinary approaches, validating efficacy, and assessing the long-term impacts. Additionally, AI-based medical technologies can aid in the prevention, prediction, and personalized management of DFU.

## AUTHOR'S CONTRIBUTIONS

The author confirms sole responsibility for the following: study conception and design, data collection, analysis and interpretation of results, and manuscript preparation.

## Figures and Tables

**Table 1:** Overview of advanced strategies for the management of DFU [107-123].

**Treatment Strategy**	**Mechanism or Process Involved**	**Reference**
**Stem Cell Therapies**		
Bone-marrow derived Mesenchymal stem cells	Secretion of various cytokines, chemokines, and growth factors, increases neo-angiogenesis	[107, 108]
Human umbilical cord Mesenchymal stem cells	reduce inflammation, regulate immunity, promote growth factors, and enhance the functions of vascular endothelial cells, fibroblasts, and keratinocytes.	[109]
Adipose-derived Mesenchymal stem cells	Angiogenesis, tissue regeneration, epithelial repair, immune modulation, inflammation reduction,	[69, 110, 111]
Placenta-derived Mesenchymal stem cells	Secreted growth factors, cytokines, and immunomodulatory substances	[112]
Amniotic fluid-derived Mesenchymal stem cells	Modulation of wound site macrophages, tissue repair, and acceleration of wound closure.	[113]
skin-derived ABCB5+ Mesenchymal stem cells	Induction of angiogenesis	[114]
Induced pluripotent stem cells	Increase angiogenesis, tissue regeneration, wound healing, and improve blood vessels improvement.	[70, 115]
**Growth Factor-based Therapies**		
Epidermal growth factor (EGF)	Tissue formation, re-epithelialization	[116-118]
Vascular endothelial growth factor (VEGF)	Inflammation, angiogenesis, increased angiogenesis, mobilizing and recruiting bone marrow-derived cells	[118, 119]
Platelet derived growth factor (PDGF)	Inflammation, re-epithelialization, collagen deposition, tissue remodelling	[118]
Hepatocyte growth factor (HGF)	Suppression of inflammation, granulation tissue formation, angiogenesis, re-epithelialization	[118]
Fibroblast growth factor (FGF)	Angiogenesis, granulation tissue formation, Re-epithelialization, and detoxification of ROS	[118]
Transforming growth factor Beta (TGFβ)	Collagen production and mechanical tensile strength of wounds, Increased accumulation of granulation tissue	[118]
Insulin-like growth factor (IGF)	Increased wound re-epithelialization, Granulation tissue formation	[118, 120]
Stromal cell-derived factor-1 (SDF-1)	Neovascularization	[121]
**Gene Therapies**		
Growth factor-based gene therapies.	Angiogenesis, granulation tissue formation, re-epithelialization, and collagen deposition	[81, 82, 122]
**Nanotechnology-based Strategies**		
Metalic nanoparticles Polymeric nanoparticles Nanomaterial-based products Nanofiber scaffolds	Angiogenesis, vascularization, epithelisation, hemostasis, cell proliferation, inflammatory modulation, and antibacterial activity	[83, 85, 123]
**Bioengineered Skin Substitutes**		
Acellular single layer Acellular Bilayer Cellular single layer Cellular Bilayer	Promotion of tissue regeneration, reduction of bacteria in the wound fluid loss, promotion of inflow of fibroblasts, neutrophils, macrophages, keratinocytes, and endothelial cells into the wound bed, secretion of the cytokines and growth factors which ultimately stimulate angiogenesis, re-epithelialization, and deposition of extracellular matrix	[73, 75, 76]
**3D Bioprinting**		
3D-bioprinted autologous minimally manipulated homologous adipose tissue	Wound healing with high-quality skin reconstruction, tissue repair, tissue regeneration	[88, 90, 91]

## LIST OF ABBREVIATIONS

ABCB5+: ATP-binding Cassette Subfamily B Member 5 Positive
AI: Artificial Intelligence
ASCs: Adipose-Derived Stem Cells
BSS: Bioengineered Skin Substitutes
DFU: Diabetic Foot Ulcer
ECM: Extracellular Matrix
EGF: Epidermal Growth Factor
ESCs: Embryonic Stem Cells
FGF: Fibroblast Growth Factor
HGF: Hepatocyte Growth Factor
IGF: Insulin-like Growth Factor
iPSCs: Induced Pluripotent Stem Cells
IWGDF: International Working Group on the Diabetic Foot
lncRNA: Long Non-coding RNA
MDR: Multi-drug Resistant
miRNA: micro-RNA
ML: Machine Learning
MMPs: Matrix Metalloproteinases
MSCs: Mesenchymal Stem Cells
PDGF: Platelet-derived Growth Factor
ROS: Reactive Oxygen Species
SDF-1: Stromal Cell-derived Factor 1
siRNA: Small Interfering RNA
TGFβ: Transforming Growth Factor Beta
VEGF: Vascular Endothelial Growth Factor
